# Complex Networks in Phase-Separating Gels: A Computer Simulation Study

**DOI:** 10.3390/polym17070880

**Published:** 2025-03-25

**Authors:** Georg Friedrich Beer

**Affiliations:** Magnetism and Interface Physics & Computational Polymer Physics, Department of Materials, ETH Zurich, 8093 Zurich, Switzerland; 6b33r@pm.me

**Keywords:** networks, gels, simulation, molecular dynamics, Lennard-Jones particles, bead-spring model, phase separation, high-density phase, morphology, surface optimization

## Abstract

The mesoscopic phase separation in two- and three-dimensional gels has been studied by computer simulation of a bead-spring model of Lennard-Jones particles. The formation of complex networks of high-density phase (HDP) has been investigated and partially explained by competing short- and long-range energies. HDP network formation was found to occur at certain combinations of temperature and spring coefficients, given sufficient particle density. The morphology of the HDP networks changed with these three parameters. HDP networks became more faceted with higher spring coefficients, wider but less dense at higher temperatures, and more voluminous and compact at larger densities. HDP network formation was preceded by a stage of HDP precipitation and followed by a stage of surface minimization.

## 1. Introduction

Polymer networks, so-called gels, can undergo two types of phase transitions: volume transitions and phase separation. This study focuses on phase separation, where the gel separates into regions with high and low polymer concentrations [1]. Up to now, research on phase separation in gels has mainly treated surface effects [2,3]. Thus, the processes occurring within the bulk of the gel are a welcome object of study for simulations, as we still lack proper methods of physical investigation [2,3]. Comprehensive understanding and prediction of microphase separation in gels could be of great use for applications where the gel’s microstructure plays a role. Two examples are the development of iongel soft solid electrolytes for solid-state batteries [4,5,6] and the comprehension of the complex deformation behavior exhibited by gels [7].

Motivated by earlier studies [8], which indicated phase separation with a characteristic length scale significantly larger than a model grid size, Peleg et al. [2] introduced a simplistic model to mimic the collection of long- and short-range forces in a gel. The model consists of particles (representing polymer density) interacting via a Lennard-Jones (LJ) potential (short-range attraction typifying polymer crystallization) connected through harmonic springs (entropy elasticity) in a two-dimensional simple square lattice structure. Their simulations revealed that, under certain circumstances, the gel phase separates into a high-density phase (HDP) and a low-density phase (LDP) with the HDP forming a filamentous, foam-like network.

In [2], Peleg et al. investigated their model for a limited set of system parameters. They quenched the gel from a simple cubic or hexagonal initial state to a few fixed temperatures, while keeping the spring constant K=1/10 (K=1/15 in the hexagonal case) and the density ρ=1/3.5 fixed (both in LJ units [9]). Peleg et al. also found that the system exhibits a hysteresis when varying the temperature. In [3], they present a phase diagram for the same constant density ρ=1/3.5, where they use the fraction of HDP particles as an order parameter. And in [10], Peleg at al. investigated networks on a random lattice.

In this paper, Peleg et al.’s [2] model is generalized to three dimensions, as suggested in [3], where Peleg et al. raised the question of whether a three-dimensional system would show linear HDP filaments connected in a network-like structure, as in two dimensions, or combine into a soap-like structure made of connected surfaces.

We want to establish measures to quantify the morphology of the evolving HDP networks and explore more of the parameter space by varying the density, spring constant, and temperature between simulations to create phase diagrams.

## 2. Materials and Methods

### 2.1. Model

In the two-dimensional elastic LJ toy model proposed and investigated by Peleg et al. [2,3,10], the gel is represented by particles initially arranged in a two-dimensional square (or hexagonal) lattice. Using LJ units [9], each particle is attributed a mass of one and is connected permanently to its four initial neighboring particles through weightless harmonic springs. These springs have a spring coefficient, denoted as *K*, and an equilibrium length of zero. Additionally, pairwise interactions between particles occur via a short-range 12-6-LJ potential, which is truncated beyond a cutoff radius $r_{c}=3\times 2^{1/6}$.

**Figure 1:**
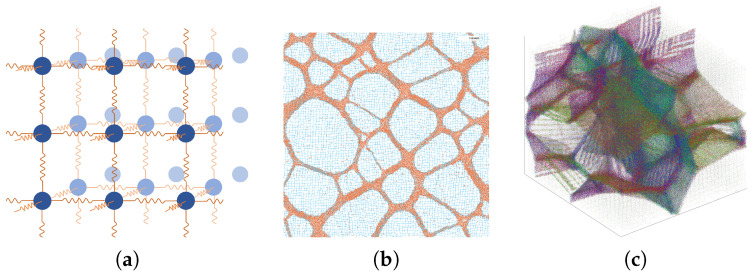
(**a**) Three-dimensional model of the gel: Simple cubic lattice of LJ particles connected with harmonic springs of equilibrium distance zero, subjected to periodic boundary conditions. (**b**) Two-dimensional gel model simulation after a time of t=4000 with density ρ=0.29, temperature T=0.3, and spring coefficient K=0.3. The springs connecting HDP particles are highlighted in orange for the graphical representation. (**c**) Three-dimensional gel model simulation after a time of t=4000 with simulation parameters ρ=0.29, T=0.3, and K=0.1. In this graphical representation, the springs are colored according to their spatial orientation. All units are dimensionless; reduced LJ units are used [9].

By employing periodic boundary conditions (PBCs), the network of harmonic springs functions to provide long-range repulsive forces, causing the particles to move apart to their simple cubic lattice positions. Conversely, the LJ forces operate at close range, attempting to “glue” the particles together. For three dimensions this model was expanded to a simple cubic lattice (Figure 1a). Here, each particle is connected permanently to its six initial neighboring particles. The dimension-dependent quantities such as distance and temperature were adapted accordingly.

### 2.2. Molecular Dynamics

The three-dimensional model was studied by a molecular dynamics (MD) simulation in the canonical ensemble. LJ units are used throughout, i.e., physical units were kept dimensionless [9]. The system was initialized as a simple cubic lattice of *N* particles and the volume of the simulation box $V=L^{3}$ was set to give the desired number density ρ=N/V. At startup, each particle was attributed a pseudo-random velocity vector v according to a Maxwell–Boltzmann (MB) distribution $f\left(v\right)∼e^{-e_{\mathrm{kin}}/T}$ with specific kinetic energy $e_{\mathrm{kin}}=v^{2}/2$, while the overall momentum was constrained to be zero. Newton’s equations of motion were integrated by a Velocity Verlet algorithm with an integration time step Δt=0.004. Temperature was kept constant by periodically rescaling the velocities every hundredth time step to the corresponding mean kinetic energy of ${\bar{E}}_{kin}^{2D}=TN$ in two dimensions and ${\bar{E}}_{kin}^{3D}=3TN/2$ in three dimensions.

### 2.3. Force Calculations

The springs were set up in a way that permanently connected a particle to the nearest periodic-boundary-condition copy of its four/six initial neighbors. In all simulations, spring lengths stayed well below one half of the simulation box length, ensuring consistency. For *d* dimensions only d×N springs had to be checked for the spring force calculations. Thus, they used only a small part of the total computing time.

The main bottleneck of these large MD simulations was the calculations of the LJ forces. With more than one hundred thousand particles, and therefore (potentially) several billion LJ force calculations per time step, it became imperative to optimize this process. This was achieved by the implementation of neighbor lists. This effectively reduced the number of LJ force calculations per time step by grouping the particles into neighborhoods. The neighborhood of a given particle was made to consist of all other particles which were likely to interact with it until the next update of the neighbor lists. In the present work, the neighborhood of a particle encompassed all other particles closer than an estimated radius:

(1) $$R_{\mathrm{NL}}=r_{c}+\Delta R\;≳\;r_{c}+12\;\Delta i_{\mathrm{NL}}\;\Delta t\;\sqrt{\frac{2T}{\pi }},$$

where $r_{c}$ denotes the cutoff radius, $\Delta i_{\mathrm{NL}}$ the number of time steps between neighbor list updates, Δt the time step, and *T* the temperature.

These neighbor lists are most effective at low temperatures facing an even particle distribution at low density. At larger temperatures, the neighborhood radius $R_{\mathrm{NL}}$ (1) increases and hence the neighborhoods encompass more particles, which reduces their effectiveness. An uneven particle distribution has a similar effect, increasing the particle count of the neighbor lists of all HDP particles drastically. It was chosen to update the list every hundredth timestep $\Delta i_{\mathrm{NL}}=100$. We checked that the criterion (1) ensures that all interactions are properly taken into account for all systems studied (see Appendix A).

As programming language and framework, MATLAB (R2017b) was used. All two-dimensional simulations were run and analyzed on a desktop PC (Intel i7-6700 CPU), the three-dimensional simulations were run on the ETH-Zurich Euler cluster. Attempts at using LAMMPS (3 Mar 2020) [11] (Versions) to simulate this model were unsuccessful.

### 2.4. Methods of Investigation

The evolving HDP networks were of a complex nature. Several methods have been found to describe the gel’s structure: (i) graphical representation, (ii) minimal particle distribution (MPD), (iii) spring length distribution (SLD), and (iv) radial shell density distribution (RSD).

(i)   Figure 1b,c show a graphical representation of the trajectories of the simulated gel. The connecting springs are drawn with a limited number of points per line. This has the effect of rendering extended springs transparent and contracted springs opaque. In two dimensions, springs smaller than $2\times 2^{1/6}$ are colored orange, highlighting the HDP. In three dimensions, foam-like structures consisting of faces and filaments evolved. To increase the visibility of these structures, each spring is colored according to its spacial orientation. Overall, this has the effect of highlighting faces and coloring them according to their orientation.
(ii)  To determine the MPD of a gel state, for each particle the distance to its closest neighbor was measured. Then, a binning algorithm was applied, with a thousand equally spaced binning intervals ranging from zero to one-fifth of the simulation box length.
(iii) To construct the SLD, this same binning algorithm was used on all current spring lengths instead of the minimal radial particle distances.
(iv) The RSD, which is the radial distribution function not normalized by the bulk density, is usually calculated by measuring all pairwise particle distances, then adding, binning, and normalizing them. With a gel of $N=60^{3}$ particles this would have meant calculating and binning $60^{6}$ distances. To keep the calculation effort reasonable, for each particle one percent of the other particles were chosen at random to then measure the distances. The binning was carried out with 2500 binning intervals over half of the simulation box length and therefore with the same binning interval length, or more precisely the same thickness of the spherical binning shell dr, as for the MPD and SLD. The resulting distribution was then normalized by the volume of the binning shell ($4\pi r^{2}dr$) and by the number of sample distances per particle (0.01×N).

**Figure 2:**
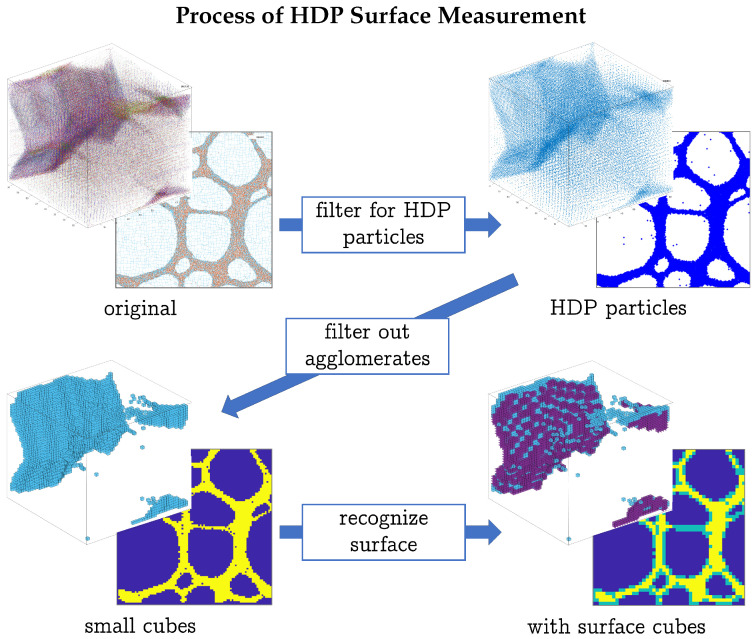
Process of surface measurement of the HDP network of a gel in two and three dimensions. First, HDP particles are determined. Then, all HDP particles which do not fall under the criterion for random agglomerates are put into small blue cubes (yellow squares). From there, the surface is beset with big violet surface cubes (green surface squares), which have the criterion of being partially comprised of small cubes/squares present.

To monitor the evolution of the structure over time, several measures were taken to express the gel’s structure in one number per time frame. This process is sketched in Figure 2. Any two particles closer to each other than a critical distance of $d_{cd}=1.1\times 2^{1/6}$ were declared *HDP particles* (see Figure 2). The simulation box was then regularly separated into *small cubes* of edge length $s_{b}=2$. To cancel out random agglomerates, such a small cube needed to encase more than four HDP particles to be declared full. Thereafter, a new grid of *big cubes* with edge length $s_{B}=2s_{b}$ was created–each big cube therefore consisted of eight small cubes in three dimensions or four in two dimensions. A big cube was now declared to be a *surface cube* if at least one, but not all, of its small cubes were full. The above values for agglomerate threshold, the size of a small box, the number of small cubes making up a big cube, and the upper and lower limits for a big cube to be a surface cube were adjusted graphically to fit the HDP structures visible by eye as well as possible.

By this algorithm, the following three numbers were obtained: the number of HDP particles, $N_{\mathrm{HDP}}$; the number of full (small) cubes, $b_{\mathrm{full}}$; and the number of (big) surface cubes, $B_{\mathrm{surf}}$. From these, five quantities of interest could be obtained for two and three dimensions *d*: the relative HDP surface area,

(2) $$a_{\mathrm{HDP}}=\frac{A_{\mathrm{HDP}}}{L^{d-1}}=\frac{1}{L^{d-1}}\times dB_{\mathrm{surf}}\times s_{B}^{d-1},$$

the fraction of HDP particles (only the particles inside of the small cubes were counted),

(3) $$n_{\mathrm{HDP}}=\frac{N_{\mathrm{HDP}}}{N},$$

the HDP volume fraction,

(4) $$v_{\mathrm{HDP}}=\frac{V_{\mathrm{HDP}}}{L^{d}}=\frac{1}{L^{d}}\times b_{\mathrm{full}}\times s_{b}^{d},$$

the density of the HDP,

(5) $$\rho_{\mathrm{HDP}}=\frac{N_{\mathrm{HDP}}}{V_{\mathrm{HDP}}}=\frac{N_{\mathrm{HDP}}}{b_{\mathrm{full}}\times s_{b}^{d}},$$

and lastly, the thickness of the HDP filaments

(6) $$\delta_{\mathrm{HDP}}=\frac{\left(d-1\right)V_{\mathrm{HDP}}}{A_{\mathrm{HDP}}}.$$

Equation (6) approximates the filament as an infinitely long cylinder, where the total surface is dominated by the curved surface area. There, the ratio of volume to surface is

(7) $$\frac{V_{\mathrm{cy}}}{A_{\mathrm{cy}}}=\frac{\pi r^{2}h}{2\pi rh}=\frac{r}{2}\;\;\mathrm{leading}\;\mathrm{to}\;\;r=\frac{2V_{\mathrm{cy}}}{A_{\mathrm{cy}}}$$

for the radius *r*. This is the ratio used in Equation (6) to obtain a rough measure of thickness.

The same argument can be made in two dimensions, taking a rectangle instead of a cylinder.

In addition to these structural measures, the total static energy of the system $E_{\mathrm{tot}}=E_{LJ}+E_{K}$ was calculated as the sum of the total pairwise LJ energy

(8) $$E_{LJ}=4\sum_{i=1}^{N}\sum_{j>i}^{N}\left(\frac{1}{r_{ij}^{12}}-\frac{1}{r_{ij}^{6}}\right)$$

and the total spring energy

(9) $$E_{K}=\frac{K}{2}\sum_{i}^{N}\sum_{j:j⌣i}^{d}r_{ij}^{2}.$$

Here, $r_{ij}$ denotes the scalar distance between particles *i* and *j*, and i⌣j symbolizes a spring connection between them.

## 3. Results

### 3.1. Evolution of an HDP Network

**Figure 3:**
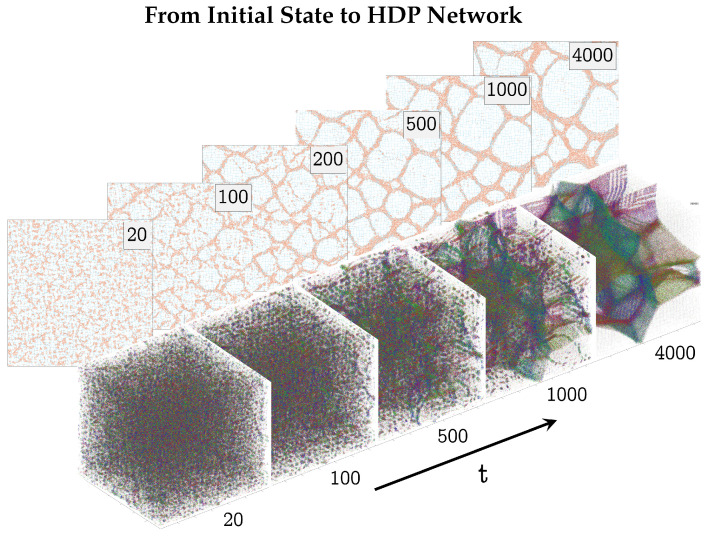
Gel simulations of the two- and three-dimensional models at different points in time *t*. In both cases, fast phase separation followed by the slower formation of a complex HDP network can be observed. *The two-dimensional* gel model was simulated with $N=100^{2}$ particles, at constant density ρ=0.3, temperature T=0.3, and spring coefficient K=0.3. The springs belonging to HDP particles are colored orange. *The three-dimensional* gel simulation was run with $N=60^{3}$ particles, at constant density ρ=0.1, temperature T=0.3, and spring coefficient K=0.3. Here, all springs are colored according to their spatial orientation. All units are dimensionless; reduced LJ units are used [9].

Under certain conditions, the gel could be observed to phase-separate into a complex network of HDP, enclosing regions of LDP. Throughout this paper, units were kept dimensionless; reduced LJ units were used [9]. Figure 3 shows this for the examples of two- and three-dimensional gels. In both examples, small HDP precipitates could be seen from the start. Over time, they grew and connected to each other to form a complex HDP network.

**Figure 4:**
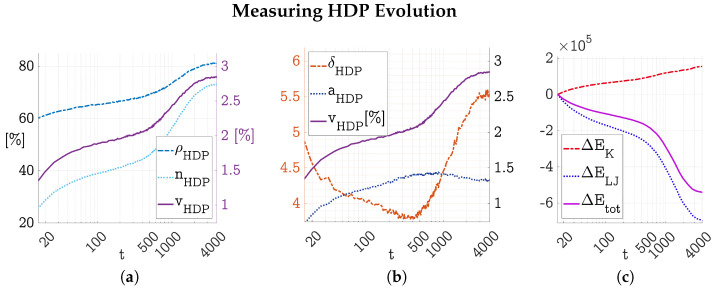
Evolution of the three-dimensional gel depicted in Figure 3 described by the measures established in Section 2.4. In the second half of the simulation, from t=2000 to t=4000, these measures approached an asymptotic state. The gel simulation was run with $N=60^{3}$ particles, at constant density ρ=0.1, temperature T=0.3, and spring coefficient K=0.3. (**a**) shows HDP density $\rho_{\mathrm{HDP}}$, HDP particle fraction $n_{\mathrm{HDP}}$, and HDP volume fraction $v_{\mathrm{HDP}}$ over time. (**b**) shows the evolution of HDP filament thickness $\delta_{\mathrm{HDP}}$ together with HDP area fraction $a_{\mathrm{HDP}}$ and HDP volume fraction $v_{\mathrm{HDP}}$ over time. And (**c**) shows the change in spring energy $\Delta E_{K}$, LJ energy $\Delta E_{\mathrm{LJ}}$, and their sum $\Delta E_{\mathrm{tot}}=\Delta E_{K}+\Delta E_{\mathrm{LJ}}$. All units are dimensionless; reduced LJ units are used [9].

To monitor this evolution, the three-dimensional gel’s trajectories were analyzed according to the measures established in Section 2.4. Figure 4a shows the particle fraction $n_{\mathrm{HDP}}$, volume fraction $v_{\mathrm{HDP}}$, and density of the HDP. All three measures showed a rapid increase at the beginning which slowed down over time until asymptotic behavior was prevalent. The final volume occupied by the HDP was a little less than 3%, concentrating 73% of all particles to a density of $\rho_{\mathrm{HDP}}=0.81$ inside the HDP.

The evolution of the relative HDP surface area $a_{\mathrm{HDP}}$ is shown in Figure 4b. Two stages can be observed. First, as the HDP precipitated and the precipitates grew larger than the agglomerate threshold, the relative HDP surface area increased. Later, after reaching a maximum of $a_{\mathrm{HDP}}=1.44$ (HDP surface area was 44% larger than one $L^{2}$ side area of the surface cube) between t=500 and t=1000, the relative HDP surface area decreased again, until transitioning to asymptotic behavior towards the end of the simulation. The relative HDP surface area $a_{\mathrm{HDP}}$ and HDP volume fraction $v_{\mathrm{HDP}}$ are linked by Equation (6) to give the HDP filament thickness $\delta_{\mathrm{HDP}}$. This HDP filament thickness decreased to a minimum just before the onset of HDP network formation was visible by eye (see Figure 3) and then increased to an asymptotic value of $\delta_{\mathrm{HDP}}=5.5$ (simulation box length was about L=130).

The evolution of the gel’s static energies showed a decrease in total LJ energy $E_{\mathrm{LJ}}$, with a lesser increase in total spring energy $E_{K}$, leading to a decrease in total static energy $E_{\mathrm{tot}}$ over the course of the simulation (see Figure 4c). After an erratic first phase, the changes in all energies slowed down to become asymptotic towards the end of the simulation.

**Figure 5:**
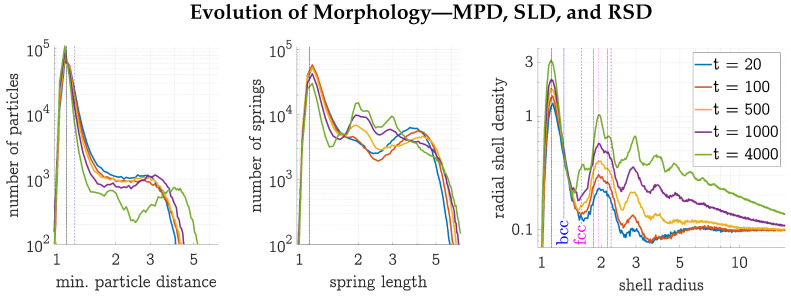
Evolution of the morphology (MPD, SLD, and RSD) of the three-dimensional gel simulation depicted in Figure 3. In all three plots, the dashed dotted black line at $2^{1/6}$ indicates the minimum of the LJ potential. The blue dashed line in the MPD at $1.1\times 2^{1/6}$ separates HDP from LDP particles. In the RSD, additional lines indicate the first three peaks of bcc and fcc structures with minimal distance of $2^{1/6}$, respectively. The gel simulation was run with $N=60^{3}$ particles at a constant density ρ=0.1, temperature T=0.3, and spring coefficient K=0.3. All units are dimensionless; reduced LJ units are used [9].

To further analyze the morphology of the evolving structure, the MPD, SLD, and RSD were examined (see Figure 5). All three distributions showed a peak around the LJ minimum, indicated by a black dashed dotted line at a distance (or length or radius) of $2^{1/6}$. The dashed blue line in the MPD separates HDP from LDP particles. Looking closely at the tip of the MPD at a distance of $d_{\mathrm{MPD}}=2^{1/6}$, a shift to lower distances can be noticed, indicating an increasing HDP density. After t=500, two peaks at $d_{\mathrm{MPD}}=1.9$ and around $d_{\mathrm{MPD}}=4$ became distinct—the right-hand-side peak moving towards larger distances indicates LDP regions of decreasing density. In the SLD, the peaks also grew more distinct over the course of the simulation. At all times, the right-hand side of the SLD showed a steep decrease in the number of large spring lengths $s_{\mathrm{SLD}}>5$. In this run, the SLD at $s_{\mathrm{SLD}}=2^{1/6}$ could be observed to decrease over time, from $5\times 10^{4}$ at the beginning to $2\times 10^{4}$ after t=4000. As more and more particles joined the HDP over time, the radial shell density grew, and its peaks became more distinct. Drawing the distances of the second to fourth closest neighbors of bcc and fcc structures with minimal distance $2^{1/6}$ into the RSD suggests that a large fraction of the HDP is fcc.

### 3.2. Parameter Variations

Originating from the parameter set of the two- and three-dimensional examples treated in Section 3.1, the parameter space spanned by the density ρ, temperature *T*, and spring coefficient *K* was further explored. The influence of ρ, *T*, and *K* could be investigated separately by choosing one of these parameters to vary while the other two were kept constant.

**Figure 6:**
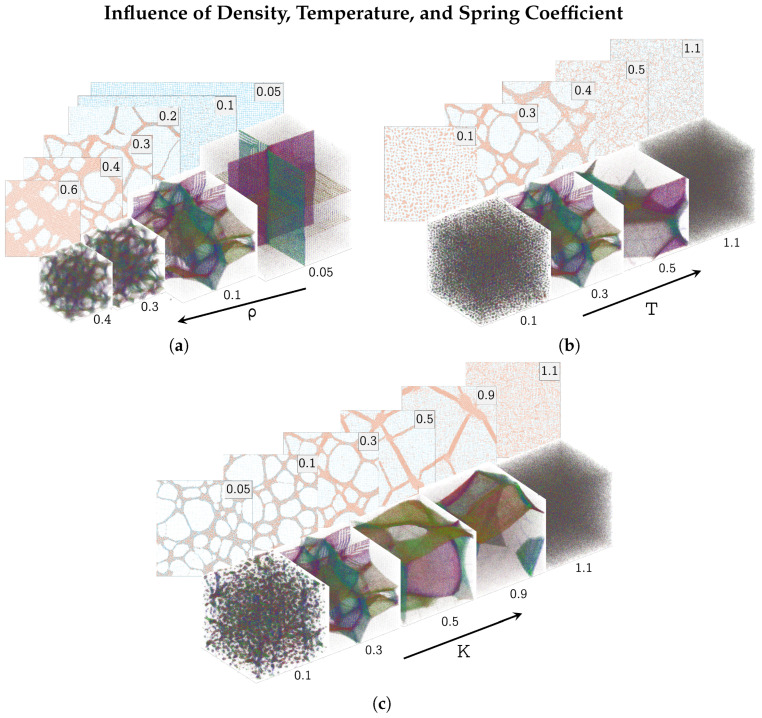
Final state (t=4000) of the gel simulations with one parameter being varied (density ρ (**a**), temperature *T* (**b**), or spring coefficient *K* (**c**)) while the other two parameters remained constant. Upper (2D) simulations were conducted with $N=100^{2}$ particles and with $\rho_{2D}=T_{2D}=K_{2D}=0.3$ if not specified. The lower (3D) simulations were conducted with $N=60^{3}$ and with constant $\rho_{3D}=0.1$, $T_{3D}=K_{3D}=0.3$ if not specified. One simulation was conducted for each set of parameters. All units are dimensionless; reduced LJ units are used [9].

The two-dimensional (2D) gel model simulations were run with $N=100^{2}$ particles, originating from $\rho_{2D}=T_{2D}=K_{2D}=0.3$; the three-dimensional (3D) simulations with $N=60^{3}$ particles, originating from $\rho_{3D}=0.1$ and $T_{3D}=K_{3D}=0.3$. Figure 6 shows the graphical representation of these simulations after a time of t=4000.

The influence of varying the density on the morphology of the end state of the simulations is shown in Figure 6a. At the lowest densities, no phase separation was observed: HDP did not precipitate. At slightly higher densities, $\rho_{2D}=0.2$ and $\rho_{3D}=0.05$, large thin structures could be observed: thin HDP filaments with large LDP holes in between in the 2D simulations; and large thin faces, normal to the initial simple cubic spring lattice, in the 3D simulations. Increasing the density further had the effect of making the HDP network bulkier, especially at the intersections of the filaments (2D simulations) or the faces (3D simulations), and of lowering the observed length scale of the network. At the highest densities, $\rho_{2D}=0.6$ and $\rho_{3D}=0.4$, HDP made up the larger part of the simulation box, with only a few LDP holes left.

At temperatures that were too low, $T_{2D}=T_{3D}\leq 0.1$, HDP precipitated but the small precipitates did not connect to a larger network (see Figure 6b). Increasing the temperatures to $T_{2D}=0.4$ and $T_{3D}=0.5$, led to an increase in the perceived length scale of the network—fewer but larger faces in the 3D simulation and larger, thicker filaments in the 2D one. Especially in the 2D simulation, the filaments appeared less compact, and fuzzier than in the ‘origin’ simulations at $T_{2D}=T_{3D}=0.3$. For larger temperatures, $T_{2D}\geq 0.5$ and $T_{3D}\geq 1$, no phase separation was observed.

If the spring coefficient *K* is zero, the springs are inactive and the gel becomes an LJ fluid instead; these have already been well researched [12,13]. Therefore, low values of K<0.05 were not of interest. At the lowest investigated spring coefficient, $K_{2D}=0.05$, of the 2D model, thin filaments could be observed, connecting chunks of HDP (see Figure 6c). The color of the observed HDP was only faintly orange, indicating the presence of many springs longer than $2\times 2^{1/6}$. The 3D gel at $K_{3D}=0.1$ showed only HDP precipitates with no apparent connections.

Moving to larger spring coefficients, $K_{2D}=0.1$, 0.3, the orange coloration of the HDP in the 2D gels became stronger, indicating more short springs inside the HDP. Additionally, the HDP filaments grew thicker, with now only slightly bulkier intersections. A similar morphology could be observed in the 3D gel at $K_{3D}=0.3$: a network of faces connected at slightly bulkier intersections.

At large spring coefficients, $K_{2D}=0.5$, 0.9, the filaments of the 2D gel became very straight, with the LDP space in between changing in shape from rounded to having sharp edges. Fewer but larger LDP areas could be seen. The LDP bubbles of the 3D gel behaved in a similar fashion: larger, but still slightly curved faces at $K_{3D}=0.5$ turning into large planar constricted faces at $K_{3D}=0.9$. For $K_{2D}\geq 1$ and $K_{3D}\geq 1$, no phase separation was observed.

### 3.3. Phase Diagrams

To create an overview of which combinations of density ρ, temperature *T*, and spring coefficient *K* phase separation and network formation could be found in the three-dimensional model, many small simulations of $N=30^{3}$ and $N=40^{3}$ particles were started and run until t=4000 ($10^{6}$ time steps), or until HDP network growth could be seen. As simulation times in three dimensions were quite high (a few weeks at this size), these simulations were limited to a plane of constant density, ρ=0.1.

**Figure 7:**
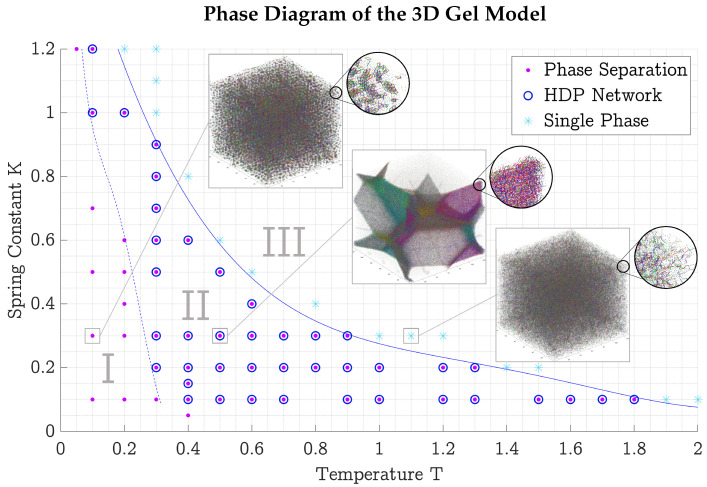
Phase diagram of the three-dimensional gel model at constant density ρ=0.1. The solid blue line separates the upper right single-phase region III from the lower left regions I and II, where phase separation into HDP and LDP was observed. The dashed blue line further divides the simulations into where HDP networks were observed (blue rings/region II) and where only HDP precipitates formed (region I). All units are dimensionless; reduced LJ units are used [9].

Figure 7 shows the phase diagram of the three-dimensional model, indicating where phase separation occurred (pink dots/regions I and II), if the HDP formed a network (blue circles/region II), or if neither thing happened (light blue stars/region III). This phase diagram is to be seen more as a collection of quenched states that include a contribution from the kinetic path than a diagram of “true” thermodynamically equilibrated states. The classification of the gel simulations into these three regions was carried out by the author, judging the graphical representation by eye.

**Figure 8:**
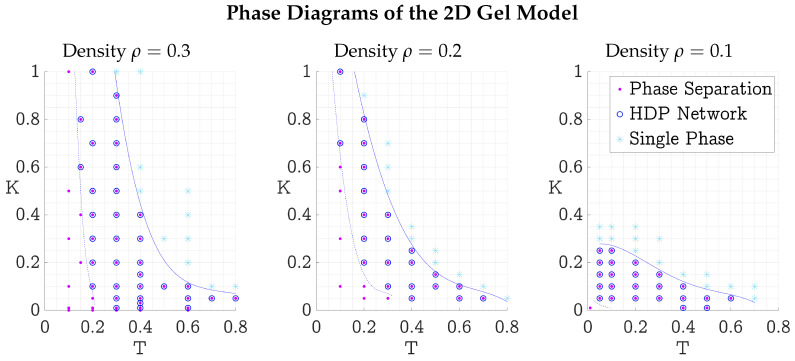
Phase diagrams of the two-dimensional gel model at constant densities: ρ=0.3, 0.2, and 0.1 from left to right. The same three regions as in the phase diagram of the three-dimensional model (Figure 7) could be observed. The solid blue line separates single-phase region III from the lower left regions I and II. The dashed blue line further divides these into region II, where HDP networks could be observed, and region I, where only HDP precipitates formed. All units are dimensionless; reduced LJ units are used [9].

The same approach was used to map the HDP network formation of the two-dimensional model. Simulations of $N=100^{2}$ and $N=150^{2}$ particles were used to investigate the gel on three planes of constant density: ρ=0.1, 0.2, and 0.3. Figure 8 shows the three resulting phase diagrams.

For all three planes of density, a similar shape of the three regions was found, with the HDP network region (II) more askew to the left in the lower-density phase diagrams. Lowering the density had a larger impact on the upper spring coefficient limit than on the temperature limit of region II. The phase separation regions (I and II) of the two-dimensional model phase diagram with ρ=0.1 were smaller than the phase separation regions of the corresponding three-dimensional model phase diagram (Figure 7).

## 4. Discussion

### 4.1. Static Energy Balance Between HDP and LDP

**Figure 9:**
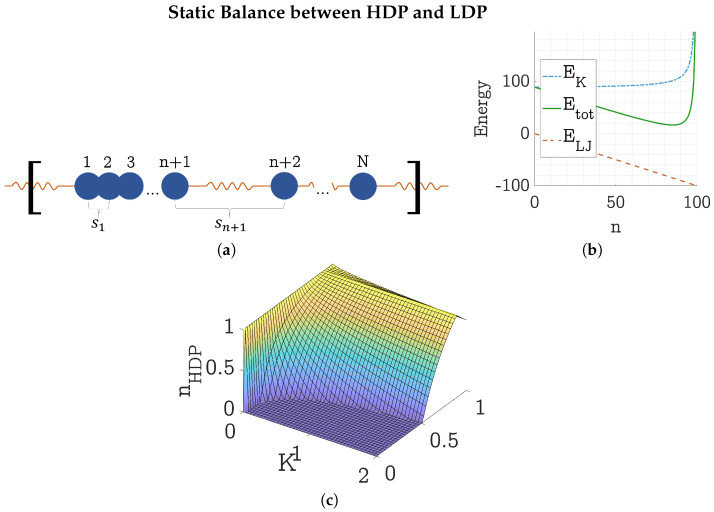
(**a**) Sketch of a one-dimensional simplification of the gel model with particles 1 to n+1 in each other’s LJ potential grouped together as HDP, and particles n+2 to *N* as LDP. The distances between the particles are denoted with $s_{n}$ for, e.g., the distance between particle *n* and particle n+1. The brackets stand for the PBC. (**b**) Quantitative plot of the total static energy $E_{\mathrm{tot}}$ of the one-dimensional model sketched in (**a**) as the sum of the LJ energy $E_{\mathrm{LJ}}$ and the harmonic spring energy $E_{K}$. Here, with N=100 particles, spring coefficient K=1, density ρ=0.75, and a box length of L=N/ρ=133. The total static energy has a minimum at n=85. (**c**) Surface of minimal total energy in the space spanned by density ρ, spring coefficient *K*, and HDP particle fraction $n_{\mathrm{HDP}}$ for the one-dimensional model sketched in (**a**). All units are dimensionless; reduced LJ units are used [9].

The phase separation of the gel can be explained by the competing LJ and harmonic energies. For this, the toy model was simplified to a one-dimensional string of *N* LJ particles connected through harmonic springs with one group of n+1 HDP particles and the rest LDP particles (see Figure 9). In this simplification, the effect of thermal vibrations is neglected—all HDP particles are assumed to be precisely in the LJ minimum at $r_{\min}=2^{1/6}$ and $E_{\min}=-1$. Therefore, all HDP springs are of equal length $s_{1}=s_{2}=\cdots =s_{n}=r_{\min}$. Further, we assume the LDP springs to be spaced equally and to be larger than the LJ cutoff radius. Thus, $s_{n+1}=s_{n+2}=\cdots =\left(L-ns_{1}\right)/\left(N-n\right)>r_{c}$.

The LJ energy of the *n* HDP particles then is $E_{\mathrm{LJ}}=\left(-1\right)\times n$, as only the LJ energy of the HDP particles is nonzero. The energy of the harmonic springs is

(10) $$\begin{array}{cc} E_{K} & =\frac{K}{2}\sum_{i=1}^{n}s_{i}^{2}+\frac{K}{2}\sum_{j=n+1}^{N}s_{j}^{2}=\frac{K}{2}\left(ns_{1}^{2}+\left(N-n\right)\frac{{\left(L-ns_{1}\right)}^{2}}{{\left(N-n\right)}^{2}}\right)\\ \; & =\frac{K}{2}\left(ns_{1}^{2}+\frac{{\left(L-ns_{1}\right)}^{2}}{\left(N-n\right)}\right). \end{array}$$

The graph of these two energies and their sum $E_{\mathrm{tot}}=E_{\mathrm{LJ}}+E_{K}$ is given in Figure 9b. For N=100 particles, a spring coefficient of K=1, a density of ρ=0.75, and a box length of L=N/ρ=133, the total static energy has a minimum at n=85.

Varying the density and spring coefficient, a surface of minimal total static energy was found—giving for each combination of ρ and *K* the optimal HDP particle fraction $n_{\mathrm{HDP}}$ (see Figure 9c). This shows an energetic motivation for phase separation, driving the system towards an equilibrium state of minimal energy.

Considering one alteration of the model, the observed growth of the network can be explained: If we imagine not one but several groups of HDP particles, for each new group created, one LJ bond is lost and replaced with a stretched spring, raising the total energy. The observed surface optimization is the reverse of this imagined process—decreasing the total energy through replacing long springs with LJ bonds.

### 4.2. Formation and Dissolution of HDP

**Figure 10:**
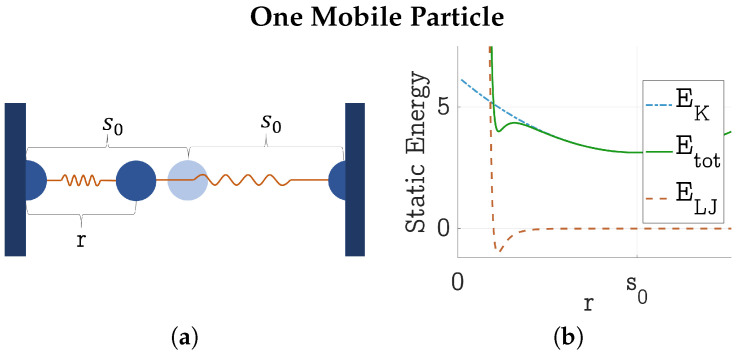
(**a**) Sketch of an LJ particle connected to two other fixed particles over harmonic springs with equilibrium length zero. The mobile particle is at a distance of $r<s_{0}$ from its left neighbor. (**b**) Harmonic spring energy $E_{K}$, LJ energy $E_{\mathrm{LJ}}$, and total energy $E_{\mathrm{tot}}$ of the mobile particle for $r<s_{0}$ with K=0.25 and $s_{0}=5$. All units are dimensionless; reduced LJ units are used [9].

To explain the influence of the temperature, and why sometimes phase separation occurred without the formation of an HDP network, let us consider another one-dimensional model (see Figure 10a): One mobile LJ particle is connected with two springs of lengths $s_{1}$ and $s_{2}$ to its neighbors, which are at a fixed distance of $2s_{0}=s_{1}+s_{2}$ from each other. A short calculation reveals the spring energy to be

(11) $$E_{K}=\frac{K}{2}\left(s_{1}^{2}+s_{2}^{2}\right)=\frac{K}{2}{\left(r^{2}+\left(2s_{0}-r\right)\right)}^{2})=K\left(r^{2}-2rs_{0}+2s_{0}\right),$$

where *r* denotes the distance of the moving particle from its left-hand-side neighbor. Together with the LJ potential, it follows that for $r<s_{0}$, the total potential of the mobile particle is

(12) $$E_{\mathrm{tot}}=K\left(r^{2}-2rs_{0}+2s_{0}\right)+4\left(\frac{1}{r^{12}}-\frac{1}{r^{6}}\right).$$

Both energies $E_{K}$ and $E_{\mathrm{LJ}}$ and the resulting total energy $E_{\mathrm{tot}}$ are plotted in Figure 10b.

It can be observed, that in order to reach the LJ minimum, which is only a local minimum in the given example, the mobile particle needs to overcome a considerable energy barrier.

If we extended this model to the right-hand side, considering a second, a third, or more mobile particles, those particles could sometimes aid this first one in reaching the left LJ minimum by approaching or by accelerating it to the left, and at other times keep it from reaching the LJ minimum by doing the opposite.

Exact calculations would need to be conducted through a statistical approach, considering the possible vibrations of the gel. For now, the intuition must suffice: It is energetically adverse for one particle to reach the other if it is moving alone. It needs more energy, through either a higher temperature or through neighboring particles vibrating collectively, which can be achieved by slightly increasing the spring coefficient. If there is too much thermal energy or if those neighbors vibrate too much, the particle is in danger of being ripped out of the LJ bond again.

This intuition can be applied to the phase diagrams found for the two- and three-dimensional gel models (see Figure 7 and Figure 8). At low temperatures and low spring coefficients, particles did not have enough vibrational energy to reach each other and form a network. Only small precipitates formed (region I). This could be remedied by reinforcing the connection between the particles by increasing the spring coefficient, to make them more likely to vibrate collectively, thus enabling the precipitates to form a network (region II). Too large spring coefficients then had the reverse effect, forcing the particles apart and dissolving the HDP again (region III). Another possibility of connecting the precipitates from region I was to increase the temperature directly, giving the particles more energy to bridge the gap between precipitates. Too large temperatures then dissolved the HDP again.

### 4.3. Future Work

Shorter simulation times, allowing more or larger simulations, would be very welcome for future projects. The most promising alternative to the rather slow MATLAB code was found in rewriting the simulation in Julia [14]. In this way, a simulation speed improvement of a factor nine could be achieved. More might be possible by improving the neighbor lists or by parallelizing the calculations.

Future works treating the same model could quantify the influence of the simulation parameters ρ, *T*, and *K* on morphology using the final measures from Section 2.4 (HDP surface fraction $a_{\mathrm{HDP}}$, HDP thickness $\delta_{\mathrm{HDP}}$, …), to move away from describing the observed change in morphology from images, as in Section 3.2. This would remove subjectivity and allow possible automatization to, e.g., find the parameters for the largest surface area.

For the same reason, fixed, non-subjective criteria should be developed for associating gels with regions I, II, and III.

One possibility would be to use the small boxes covering the HDP volume (established in Section 2.4). Region II could be defined as a percolating structure of small boxes. This could be implemented with a forest fire algorithm like in [15,16]. Other suitable criteria could be found, taking a certain minimal HDP particle fraction combined with a minimal HDP thickness. Either would enable an automated mapping of phase diagrams like Figure 7 and Figure 8.

Instead of running simulations with constant parameters, outgoing from an initial state, simulations could be started from the final/asymptotic state of a simulation of other parameters. This would serve to further investigate the temperature hysteresis Peleg et al. [2] found to see if a hysteresis could also be observed in the other two parameters (ρ and *K*), and if the same phase diagram would develop eventually.

The prediction for the optimal HDP particle fraction of a one-dimensional gel model (Section 4.1) could be compared to simulations of a one-dimensional gel model, and generalized to also include the influence of temperature.

To come closer to reality, the model could be altered by, e.g., using a distribution of spring coefficients instead of a uniform *K* or by randomizing the structure through, e.g., cutting/disabling some of the springs of the initial simple cubic lattice, as in [10]. Another approach would be to insert a second species of solvent particles.

The two-dimensional model of Peleg at al. [2,3] has already been compared to experimental results from investigating biopolymer mixtures in confined geometries [17] and found to be “in good agreement” with their findings.

Another direct link to observable gels might be achieved through changing the three-dimensional model to simulate a surface, by changing one dimension from periodic to non-periodic with a boundary against vacuum or an LJ fluid. The resulting surface could then be compared to scanning electron microscopy (SEM) images or atomic force microscopy (AFM) images of gel surfaces, as obtained in [18,19,20,21].

Alternatively, the structure resulting from larger three-dimensional simulations could be compared to gels investigated with micro-computed tomography (micro-CT) [22].

In [3], Peleg et al. investigated the shear modulus of a two-dimensional gel with an HDP network. This work could be continued (also for three dimensions) with an effort to link the system parameters over the gel morphology to its resulting mechanical properties.

## 5. Conclusions

The generalization of the gel model introduced by Peleg et al. [2] to three dimensions has shown structures which lie somewhere between their two hypotheses. The HDP formed a soap-like structure made of connected surfaces, but depending on the system parameters, the HDP also showed tendencies to form linear filament network-like structures, where the faces connected.

We systematically explored the (ρ,K,T) parameter space for the two- and three-dimensional gel model, sketching the shape of regions with HDP network formation in four phase diagrams (see Figure 7 and Figure 8). Then, in Section 2.4 we established measures allowing quantitative characterization of the gel’s structure.

In Section 4.1 and Section 4.2, the phase separation occurring in the gel model has been explained and discussed through two one-dimensional simplifications. Section 4.1 explains why there exists an optimal fraction of HDP particles in a static equilibrium and Section 4.2 attempts to justify the shape of the found phase diagrams with a dynamic approach.

We believe that future work on this model should try to connect it to real gels to see if this gel model can be used to predict the morphology of laboratory-produced gels and in this way this help adjust the gel’s material properties by tuning its microstructure. 

## Data Availability

Trajectory-data available at ETH research collection (https://www.research-collection.ethz.ch/handle/20.500.11850/728415, accessed on 10 March 2025).

## Appendix A

For a given particle, the neighbor list needs to contain at least all particles in a radius $R_{\mathrm{NL}}=r_{c}+\Delta R$ that could interact with it in a given time interval $\Delta t\cdot \Delta i_{\mathrm{NL}}$. Where the list is updated every $\Delta i_{\mathrm{NL}}$-th time step and $r_{c}=3\times 2^{1/6}$ is the cutoff radius above which the LJ potential is set to zero. A reasonable $R_{\mathrm{NL}}$ can be estimated as follows. Considering the MB distribution of the velocities, it is very unlikely for particles to have velocities exceeding $4\;\bar{v}$. The probability for that is $p\left(v\geq 4\;\bar{v}\right)\leq 10^{-8}$. Accounting for the relative movement of the particles with a factor of two, this gives

(A1) $$\Delta R>2\times 4\bar{v}\times \Delta i_{\mathrm{NL}}\;\Delta t,$$

where the mean speed is given by $\bar{v}=\sqrt{8T/\pi }$, which yields Equation (1) as an estimate for the radius of the neighbor lists, ensuring that all LJ interactions are properly taken into account.
